# Supplementary material for: Morphological convergence in ‘river dolphin’ skulls
Source: PeerJ. 2017 Nov 21;5:e4090. doi: 10.7717/peerj.4090 (PMC5701545; doi:10.7717/peerj.4090)
Supplement: Supplemental Information 1 — Figure S1. Phylogeny of the Odontoceti, adapted from Steeman et al. (2009). Genera in blue represent those included in this study. River dolphin genera are highlighted by the use of cartoons depicting each species. Table S1. Accession numbers and species of specimens sampled. Taxonomic names follow that of Rice (1998). [file peerj-05-4090-s001.docx]

**Supplemental Information S1**

**Specimens used**

Below are the full details of the specimens used and their accession numbers. All specimen data are available from the Natural History Museum’s Data Portal at <http://dx.doi.org/10.5519/0082274> (Page & Cooper 2017) (Table S1).

Table S1**. Accession numbers and species of specimens sampled.** Taxonomic names follow that of Rice (1998).

| Accession number | Species | Cranium | Mandible | Both |
| --- | --- | --- | --- | --- |
| NHMUK_1933.10.13.2 | Delphinapterus_leucas | x |  |  |
| NHMUK_1933.10.13.4 | Delphinapterus_leucas |  |  | x |
| NHMUK_1933.10.13.1 | Delphinapterus_leucas | x |  |  |
| NHMUK_1952.10.30.2 | Delphinapterus_leucas | x |  |  |
| NHMUK_SW.1932.60 | Delphinapterus_leucas | x |  |  |
| NHMUK_1973.107 | Delphinus_delphis |  | x |  |
| NHMUK_1888.5.20.2 | Delphinus_delphis |  |  | x |
| NHMUK_1946.12.24.3 | Delphinus_delphis |  |  | x |
| NHMUK_1973.106 | Delphinus_delphis |  |  | x |
| NHMUK_1946.12.24.1 | Delphinus_delphis |  |  | x |
| NHMUK_SW.1927.25 | Grampus_griseus |  |  | x |
| NHMUK_SW.1929.7 | Grampus_griseus |  |  | x |
| NHMUK_SW.1933.14 | Grampus_griseus |  |  | x |
| NHMUK_SW.1938.7 | Grampus_griseus |  |  | x |
| NHMUK_SW.1940.6 | Grampus_griseus | x |  |  |
| NHMUK_GERM 1169b | Inia_geoffrensis |  |  | x |
| NHMUK_1856.8.2.1 | Inia_geoffrensis |  |  | x |
| NHMUK_1937.5.26.1 | Inia_geoffrensis |  |  | x |
| NHMUK_1939.5.13.1 | Inia_geoffrensis |  |  | x |
| NHMUK_SW.1926.17 | Lagenorhynchus_acutus |  |  | x |
| NHMUK_SW.1929.25 | Lagenorhynchus_acutus |  |  | x |
| NHMUK_1920.6.28.1 | Lagenorhynchus_acutus |  |  | x |
| NHMUK_1944.11.30.1 | Lagenorhynchus_australis |  |  | x |
| NHMUK_1961.6.12.1 | Lagenorhynchus_australis |  |  | x |
| NHMUK_1944.11.16.1 | Lagenorhynchus_obscurus |  |  | x |
| NHMUK_1944.11.16.2 | Lagenorhynchus_obscurus |  |  | x |
| NHMUK_1949.5.30.1 | Lagenorhynchus_obscurus |  |  | x |
| NHMUK_1922.6.22.1 | Lipotes_vexillifer |  |  | x |
| NHMUK_1908.2.28.1 | Mesoplodon_bidens |  | x |  |
| NHMUK_1920.12.20.1 | Mesoplodon_bidens |  | x |  |
| NHMUK_SW.1964.6.3.7 | Mesoplodon_bidens | x |  |  |
| NHMUK_1949.8.19.1 | Mesoplodon_hectori |  |  | x |
| NHMUK_1937.10.30.2 | Monodon_monoceros | x |  |  |
| NHMUK_1885.2.20.1 | Monodon_monoceros | x |  |  |
| NHMUK_1937.10.30.1 | Monodon_monoceros | x |  |  |
| NHMUK_1949.11.2.1 | Monodon_monoceros | x |  |  |
| NHMUK_1889.8.6.1 | Neophocaena_phocaenoides | x |  |  |
| NHMUK_1902.6.10.65 | Neophocaena_phocaenoides |  |  | x |
| NHMUK_1903.9.12.3 | Neophocaena_phocaenoides |  |  | x |
| NHMUK_1950.9.19.1 | Phocoena_phocoena |  | x |  |
| NHMUK_SW.1928.49 | Phocoena_phocoena |  | x |  |
| NHMUK_SW.1928.49 | Phocoena_phocoena | x |  |  |
| NHMUK_SW.1933.50 | Phocoena_phocoena | x |  |  |
| NHMUK_1957.6.4.1 | Phocoenoides_dalli |  |  | x |
| NHMUK_1965.1.19.2 | Phocoenoides_dalli |  |  | x |
| NHMUK_1843.8.18.5 | Platanista_gangetica | x |  |  |
| NHMUK_1874.6.1.1 | Platanista_gangetica |  |  | x |
| NHMUK_1884.3.29.1 | Platanista_gangetica |  |  | x |
| NHMUK_1884.3.29.1 | Platanista_gangetica |  | x |  |
| NHMUK_GERM 344a | Platanista_gangetica |  |  | x |
| NHMUK_1866.12.13.1 | Pontoporia_blainvillei | x |  |  |
| NHMUK_1925.11.21 | Pontoporia_blainvillei |  |  | x |
| NHMUK_1939.4529 | Pontoporia_blainvillei |  |  | x |
| NHMUK_1961.6.14.4 | Pseudorca_crassidens |  |  | x |
| NHMUK_1961.6.14.86 | Pseudorca_crassidens |  |  | x |
| NHMUK_1961.6.14.27 | Pseudorca_crassidens |  |  | x |
| NHMUK_1984.1759 | Sousa_chinensis |  |  | x |
| NHMUK_1957.5.9.7 | Stenella_attenuata |  |  | x |
| NHMUK_1959.12.13.2 | Stenella_attenuata |  |  | x |
| NHMUK_1959.12.31.5 | Stenella_attenuata |  |  | x |
| NHMUK_1869.5.21.2 | Stenella_attenuata |  |  | x |
| NHMUK_1882.1.2.1 | Stenella_clymene |  |  | x |
| NHMUK_1948.3.13.2 | Stenella_clymene | x |  |  |
| NHMUK_1954.9.9.5 | Stenella_clymene |  |  | x |
| NHMUK_1992.92 | Stenella_clymene |  |  | x |
| NHMUK_1970.1509 | Stenella_clymene |  |  | x |
| NHMUK_1938.2.5.1 | Stenella_coeruleoalba |  |  | x |
| NHMUK_1961.5.31.1 | Stenella_coeruleoalba |  |  | x |
| NHMUK_SW.1934.1 | Stenella_coeruleoalba |  |  | x |
| NHMUK_1949.1.7.2 | Steno_bredanensis |  |  | x |
| NHMUK_1952.8.1.1 | Steno_bredanensis |  |  | x |
| NHMUK_GERM 345e | Steno_bredanensis |  |  | x |
| NHMUK_GERM 345f | Steno_bredanensis |  |  | x |
| NHMUK_1954.6.25.2 | Steno_bredanensis |  |  | x |
| NHMUK_1949.10.27.3 | Tursiops_aduncus |  |  | x |
| NHMUK_1954.9.9.4 | Tursiops_aduncus |  |  | x |
| NHMUK_1970.1512 | Tursiops_aduncus |  |  | x |
| NHMUK_1964.9.2.2 | Tursiops_aduncus |  |  | x |
| NHMUK_1952.7.30.3 | Tursiops_truncatus |  |  | x |
| NHMUK_1952.7.30.6 | Tursiops_truncatus |  |  | x |
| NHMUK_1960.5.11.10 | Tursiops_truncatus |  |  | x |
| NHMUK_1960.5.11.7 | Tursiops_truncatus |  |  | x |
| NHMUK_1960.5.11.9 | Tursiops_truncatus |  |  | x |

Figure S1. **Phylogeny of the Odontoceti, adapted from Steeman *et al.* (2009).** Genera in blue represent those included in this study. River dolphin genera are highlighted by the use of cartoons depicting each species.


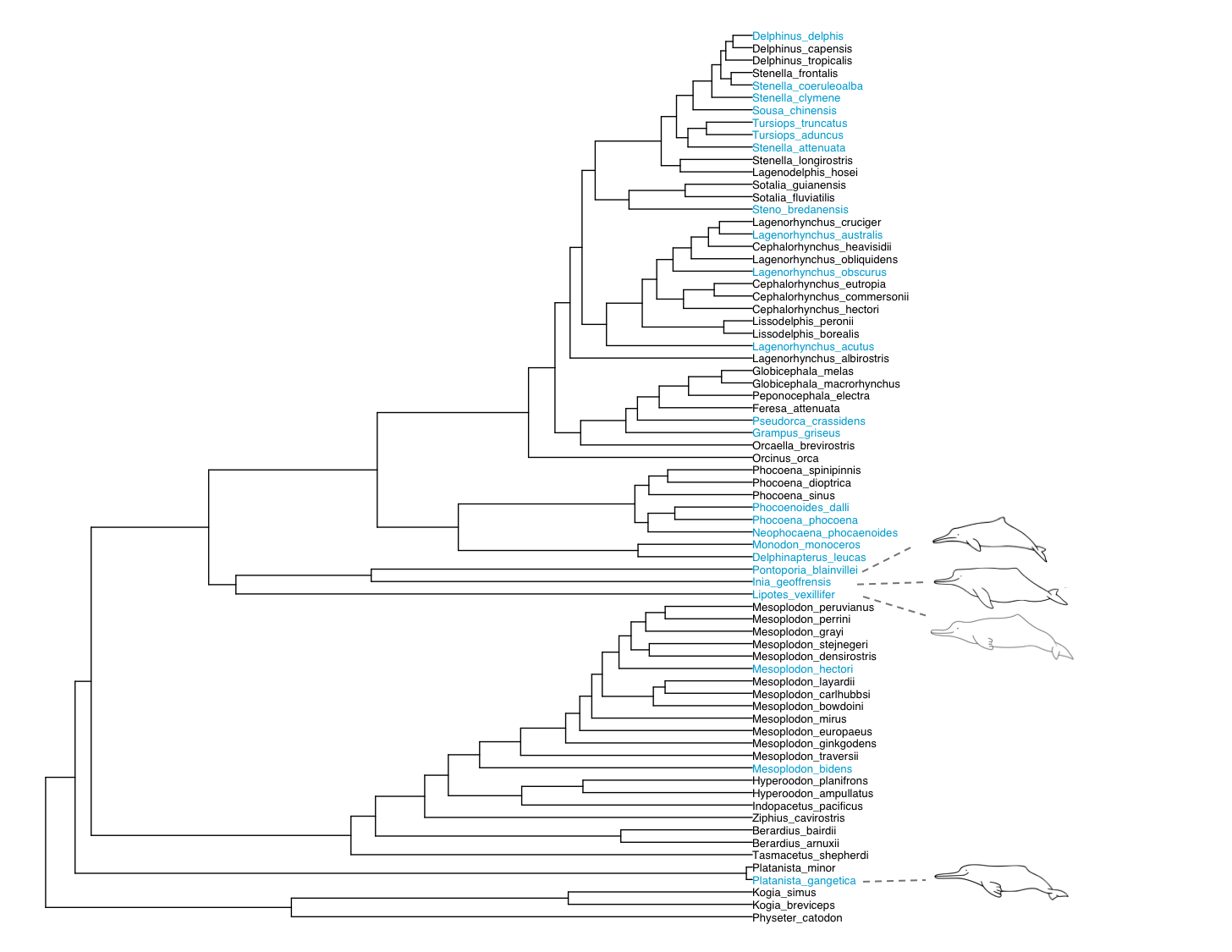


**References**

Page EC, and Cooper N. 2017. Dataset: Crania and mandible data from 'river dolphins' and other odontocetes. Natural History Museum Data Portal (data.nhm.ac.uk). <http://dx.doi.org/10.5519/0082274>.

Rice C. 1998. Marine mammals of the world, systematics and distributuion. *Society for Marine Mammalogy Special Publications* 4:1-231.

Steeman ME, Hebsgaard MB, Fordyce RE, Ho SY, Rabosky DL, Nielsen R, Rahbek C, Glenner H, Sørensen MV, and Willerslev E. 2009. Radiation of extant cetaceans driven by restructuring of the oceans. *Systematic Biology* 58:573-585.
