# Supplementary material for: Morphological convergence in ‘river dolphin’ skulls
Source: PeerJ. 2017 Nov 21;5:e4090. doi: 10.7717/peerj.4090 (PMC5701545; doi:10.7717/peerj.4090)
Supplement: Supplemental Information 2 — Table S2. Descriptions of the landmarks (points) and curves (semi-landmarks), digitised on the images of crania in ventral view for all specimens. Table S3.Descriptions of the landmarks (points) and curves (semi-landmarks), digitised on the images of mandibles in the dorsal view for all specimens. Table S4. Mean percentage of ‘true length’ (200 equally spaced points) represented by resampling of points on the cranial curves. The number of semi-landmark points we used are highlighted in bold. Table S5.Mean percentage of ‘true length’ (200 equally spaced points) represented by resampling of points on the mandibular curves. The number of semi-landmark points we used are highlighted in bold. [file peerj-05-4090-s002.docx]

**Supplemental Information S2**

**Landmark descriptions**

Below is further information describing details of the landmarks we used to summarise morphological shape of the skulls of the Odontoceti specimens sampled. Landmark numbers and curves refer to Figure 2 in the main paper (example on *Tursiops truncates*, Figure 2B and D, and *Pontoporia blainvillei*, Figure 2A and C). Tables S2 and S3 describe the landmarks used in the analysis. The numbers correspond to numbers in Figure 2 of the main paper.

To determine the number of semi-landmarks to sample for each curve we followed the protocol of MacLeod (2012). This simple approach measures points spaced on a drawn curve in an arbitrary manner. A dense sample of points is first placed around the curve. The length of this curve is then taken to be the true length of the curve. We then used interpolation methods to reduce this set of points till we reached the minimum amount of points that could account for at least 95% of the ‘true length’ of the curve. We did this by first resampling 200 points on each curve in TPSdig (Rohlf 2006) on a subset of specimens which described most of the variation in the dataset (i.e. one species from each family) and recorded this length as the true length. We then reduced the number of points till we reached 95% accuracy of the true length of the curve or to the minimum number of semi-landmarks possible whilst still having to mark the beginning and end of the curve (i.e. three). The number of points samples and the % of the true length they explained are shown in Table S4 and S5.

**Error Analyses**

The placement, and subsequent orientation of specimens for photography is one of the main sources of error in 2D GMM studies in addition to landmark placement and repeatability (Fruciano 2016; Zelditch et al. 2012). To measure error in photographing (and presentation of specimens), we photographed three specimens of the same species (*Tursiops aduncus*), three times for each view (ventral cranium and dorsal mandible), cycling through the three specimens each time, so the specimen had to be removed and re-positioned after every photograph. To measure error in digitising, we then digitised landmarks on all of these images three times, cycling through each set of repeats each time. We then performed a nested Procrustes ANOVA, where replicate was nested within individual using the function ‘procD.lm’ in geomorph (Adams et al. 2017) on GPA coordinates.

Using the equations outlined below by Fruciano (2016), we then calculated repeatability for both the cranium and mandible datasets.

$$R={S_{A}^{2}}/{(S_{W}^{2}}+ S_{A}^{2})$$

Where $S_{A}^{2}$ is the among-individuals variance component and $S_{W}^{2}$is the within-individuals variance component and R is repeatability. $S_{A}^{2}$and $S_{W}^{2}$can be computed as:

$$S_{A}^{2}= {({MS}_{among}- {MS}_{within})}/n$$

$$S_{W}^{2}= {MS}_{within}$$

Where n is the number of repeated measurements while ${MS}_{among}$ and ${MS}_{within}$ are, respectively, the among-groups and within-groups ANOVA sum of squares (Fruciano 2016).

Repeatability in the cranial data set was 91.3% and in the mandibular data set was 93.1%. This gave us confidence in the precision of our photographing and digitising, and that our results are not due to measurement error.

Table S2**. Descriptions of the landmarks (points) and curves (semi-landmarks), digitised on the images of crania in ventral view for all specimens.**

| Landmarks | Description |
| --- | --- |
| 1 | Rostral tip. |
| 2 + 3 | Left (2) and right (3) intersection between the maxilla and lacrimojugal bones. |
| 4 + 5 | Left (4) and right (5) anteriormost points on the lacrimojugal. |
| 6 + 7 | The most anterior point on the left (6) and right (7) zygomatic arch. |
| 8 + 9 | Left (8) and right (9) most posterior point of the zygomatic arch, placed outside of the brain case. |
| 10 + 11 | Left (10) and right (11) ventral most point on the paraoccipital process. |
| 12 | Midway point between the occipital condyles. |
| Curve C1  (5 equally spaced points) | Outline of the zygomatic arch on the left beginning at landmark 9, and ending at 7. |
| Curve C2  (5 equally spaced points) | Outline of the zygomatic arch on the left beginning at landmark 10, and ending at 8. |
| Curve C3  (3 equally spaced points) | Outline of the left side of the palate, excluding dental structures, beginning at landmark 2, ending at landmark 1. |
| Curve C4  (3 equally spaced points) | Outline of the left side of the palate, excluding dental structures, beginning at landmark 3, ending at landmark 1. |

Table S3. **Descriptions of the landmarks (points) and curves (semi-landmarks), digitised on the images of mandibles in the dorsal view for all specimens.**

| Landmarks | Description |
| --- | --- |
| 1 | Most anterior point of the mandible. |
| 2 | Centroid of the posterior-most point of fusion between the two mandibles. |
| 3 + 4 | Left (3) and right (4) posterior point of the alveolar groove. |
| 5 + 6 | Left (5) and right (6) interior most points on the mandibular condyle. |
| 7 + 8 | Left (7) and right (8) exterior most point of the mandibular condyle. |
| Curve C5  (3 equally spaced points) | Outline of the left mandible excluding dental structures, starting at landmark 7, ending at landmark 1. |
| Curve C6  (3 equally spaced points) | Outline of the right mandible excluding dental structures, starting at landmark 8, ending at landmark 1. |

Table S4**. Mean percentage of ‘true length’ (200 equally spaced points) represented by resampling of points on the cranial curves.** The number of semi-landmark points we used are highlighted in bold.

|  | % length described | |
| --- | --- | --- |
| # of Points | Curve C1 | Curve C2 |
| 7 | 97.2 | 97.1 |
| **5** | **95.1** | **94.9** |
| 4 | 91.9 | 92.0 |
|  | Curve C3 | Curve C4 |
| 14 | 99.3 | 99.2 |
| 4 | 98.2 | 98.1 |
| **3** | **98.0** | **97.9** |

Table S5. **Mean percentage of ‘true length’ (200 equally spaced points) represented by resampling of points on the mandibular curves.** The number of semi-landmark points we used are highlighted in bold.

|  | % length described | |
| --- | --- | --- |
| # of Points | Curve C5 | Curve C6 |
| 30 | 99.6 | 99.5 |
| 15 | 99.2 | 99.0 |
| 7 | 98.8 | 98.6 |
| **3** | **98.6** | **98.4** |

References

Adams D, Collyer M, Kaliontzopoulou A, and Sherratt E. 2017. Geomorph: Software for geometric morphometric analyses. *R package version 3.0.5*.

Fruciano C. 2016. Measurement error in geometric morphometrics. *Development Genes and Evolution* 226:139-158.

MacLeod N. 2012. Going Round the Bend II: Extended Eigenshape Analysis. *Available at* [http://www.palass.org/](http://www.palass.org) (accessed 12 Febuary 2017).

Rohlf F. 2006. tpsDig program, Version 2.10. *Department of Ecology and Evolution, State University of New York, Stony Brook*.

Zelditch ML, Swiderski DL, and Sheets HD. 2012. *Geometric morphometrics for biologists: a primer*: Academic Press.
