# Supplementary material for: Morphological convergence in ‘river dolphin’ skulls
Source: PeerJ. 2017 Nov 21;5:e4090. doi: 10.7717/peerj.4090 (PMC5701545; doi:10.7717/peerj.4090)
Supplement: Supplemental Information 3 — Table S6. The eigenvalues, proportion of variance and highest loading landmarks (<−0.2 or >0.2) for the PCs that represent >95% of the total variance in the cranium and mandible dataset. Table S7. Principal component coefficients (eigenvectors) describing the loadings of each landmark’s x and y position for the PCs that represent <95% of the total variation in the cranium dataset. Landmarks that have a loading <−0.2 or >0.2 are highlighted in bold. Table S8. Principal component coefficients (eigenvectors) describing the loadings of each landmark’s x and y position for the PCs that represent >95% of the total variation in the mandible dataset. Landmarks that have a loading <−0.2 or >0.2 are highlighted in bold. Table S9.Results of phylogenetic ANOVAs on the major (>95%) PC axes for both the cranium and mandible datasets. Values in bold are significant, df indicates degrees of freedom. Table S10.Results of ANOVAs on the major (>95%) PC axes for both the cranium and mandible datasets using specimen-level data. Values in bold are significant, df indicates degrees of freedom. [file peerj-05-4090-s003.docx]

**Supplemental Information S3**

**Supplemental Principal Component Analysis Results**

Here we present supplemental Principal Component Analysis (PCA) results, describing the variance explained by each major PC (>95% variation) and eigenvalues (Table S6). The loadings of the landmarks on each major PC axis (eigenvectors) are presented in Tables S7 and S8.

Table S6**. The eigenvalues, proportion of variance and highest loading landmarks (< -0.2 or > 0.2) for the PCs that represent >95% of the total variance in the cranium and mandible dataset.**

| Principal Component | Eigenvalue | % Variance | Cumulative Variance % | Highest loading landmarks |
| --- | --- | --- | --- | --- |
| cranium |  |  |  |  |
| 1 | 0.0149 | 83.0 | 83.0 | 1 |
| 2 | 0.00155 | 8.65 | 91.7 | 2,3,6,7,10,11 |
| 3 | 0.000473 | 2.63 | 94.3 | 2,3,12 |
| 4 | 0.000251 | 1.40 | 95.7 | 2,3,5,10,12 |
| mandible |  |  |  |  |
| 1 | 0.0208 | 47.0 | 47.0 | 1,2,3,4 |
| 2 | 0.0159 | 36.1 | 83.1 | 1,2,3,4 |
| 3 | 0.00660 | 14.8 | 97.9 | 2,3,4,5,6,7,8 |

| Landmark | PC1 | PC2 | PC3 | PC4 |
| --- | --- | --- | --- | --- |
| 1x | **-0.740** | 0.129 | 0.103 | -0.0654 |
| 1y | -0.000735 | 0.0513 | 0.00299 | 0.0894 |
| 2x | 0.103 | **0.278** | **-0.221** | **0.373** |
| 2y | 0.136 | 0.162 | 0.190 | **-0.233** |
| 3x | 0.115 | **0.218** | **-0.201** | **0.433** |
| 3y | -0.144 | -0.173 | -0.136 | 0.174 |
| 4x | 0.145 | 0.164 | -0.118 | -0.0954 |
| 4y | 0.122 | 0.0151 | 0.284 | 0.0762 |
| 5x | 0.165 | 0.151 | -0.179 | -0.127 |
| 5x | -0.123 | 0.00686 | -0.276 | **-0.216** |
| 6x | 0.0545 | **-0.435** | 0.0799 | -0.0414 |
| 6y | 0.162 | 0.118 | 0.159 | -0.0142 |
| 7x | 0.0608 | **-0.476** | 0.0172 | -0.0257 |
| 7y | -0.160 | -0.147 | -0.227 | 0.00363 |
| 8x | 0.100 | 0.111 | 0.0102 | -0.126 |
| 8y | 0.146 | -0.167 | -0.0482 | -0.0832 |
| 9x | 0.118 | 0.121 | -0.0379 | -0.099 |
| 9y | -0.146 | 0.140 | 0.0113 | 0.116 |
| 10x | 0.0266 | **0.265** | 0.147 | -0.142 |
| 10y | 0.171 | 0.008 | -0.114 | **0.278** |
| 11x | 0.0230 | **0.228** | 0.0964 | -0.173 |
| 11y | -0.170 | 0.0121 | 0.141 | -0.149 |
| 12x | -0.0958 | 0.0699 | **0.618** | **0.362** |
| 12y | 0.00323 | 0.0453 | 0.0422 | 0.0342 |

Table S7**. Principal component coefficients (eigenvectors) describing the loadings of each landmark’s x and y position for the PCs that represent >95% of the total variation in the cranium dataset.** Landmarks that have a loading < -0.2 or > 0.2 are highlighted in bold.

Table S8**. Principal component coefficients (eigenvectors) describing the loadings of each landmark’s x and y position for the PCs that represent >95% of the total variation in the mandible dataset.** Landmarks that have a loading < -0.2 or > 0.2 are highlighted in bold.

| Landmark | PC1 | PC2 | PC3 |
| --- | --- | --- | --- |
| 1x | **0.600** | **0.237** | 0.00627 |
| 1y | 0.00735 | 0.00125 | -0.0174 |
| 2x | **-0.242** | **-0.733** | **0.449** |
| 2y | -0.00317 | 0.00243 | -0.0109 |
| 3x | **-0.480** | **0.387** | 0.157 |
| 3y | -0.0365 | -0.150 | **-0.203** |
| 4x | **-0.482** | **0.387** | 0.168 |
| 4y | **0.0475** | 0.141 | **0.204** |
| 5x | 0.0171 | -0.0884 | -0.161 |
| 5x | -0.14 | -0.0410 | **-0.336** |
| 6x | 0.0123 | -0.0859 | -0.115 |
| 6y | 0.142 | 0.0435 | **0.347** |
| 7x | 0.0110 | -0.105 | -0.184 |
| 7y | -0.161 | -0.0240 | **-0.369** |
| 8x | 0.0111 | -0.0915 | -0.148 |
| 8y | 0.159 | 0.0327 | **0.375** |

**Testing for convergence - species level**

Here we present the results of *post hoc* ANOVAs on major PC axis (Table S9). These tests whether the four river dolphin genera occupy significantly different positions on the major axes of shape variation.

Table S9. **Results of phylogenetic ANOVAs on the major (>95%) PC axes for both the cranium and mandible datasets.** Values in bold are significant, df indicates degrees of freedom.

| Principal  Component | df | F-statistic | P-value |
| --- | --- | --- | --- |
| cranium |  |  |  |
| PC1 | 1,22 | 13.9 | 0.078 |
| PC2 | 1,22 | 12.09 | 0.097 |
| PC3 | 1,22 | 0.349 | 0.777 |
| PC4 | 1,22 | 0.561 | 0.765 |
| mandible |  |  |  |
| **PC1** | **1,21** | **19.3** | **< 0.05** |
| PC2 | 1,21 | 8.53 | 0.169 |
| PC3 | 1,21 | 2.45 | 0.475 |

**Specimen-level analyses**

Our analyses use species mean GPA coordinates for all analyses. However, this removes a lot of the variation seen within species, therefore we repeated our analyses at the specimen-level. The methods and results are described below.

**Exploring shape variation in dolphin skulls - specimen level**

We repeated the PCA using specimen-level rather than species-level GPA coordinates. The first five PC scores explain 95.3% of the shape of odontocete crania, whilst the first three PC scores explain 97.6% of variation in the shape of odontocete mandibles.

**Testing for convergence - specimen level**

To determine whether ‘river dolphins’ have significantly different skull shapes compared to other odontocetes, we performed a Procrustes ANOVA on the specimen GPA coordinates for both cranial and mandibular datasets using the function ‘procD.lm’ in geomorph (Adams et al. 2017) with 1000 iterations and residual randomization for significance testing. The ‘river dolphins’ have significantly different skulls shapes compared to other odontocetes for both the cranium (Procrustes ANOVA: F1,75 = 34.7, p < 0.001) and mandible (ANOVA: F1,65 = 32.5, p < 0.001) datasets.

Results of *post hoc* ANOVAs for individual major PCs can be found in Table S10. They show that ‘river dolphin’ specimens have significantly different skulls shapes compared to other odontocete specimens for both the cranium (p < 0.001) and mandible (p < 0.001) datasets, and river dolphin specimens occupy significantly different positions on cranial PC1 (p < 0.001) and PC2 (p < 0.001) and mandibular PC1 (p < 0.001) and PC2 (p < 0.001) compared to other odontocete specimens.

Table S10. **Results of ANOVAs on the major (>95%) PC axes for both the cranium and mandible datasets using specimen-level data.** Values in bold are significant, df indicates degrees of freedom**.**

| Principal component | df | F-statistic | P-value |
| --- | --- | --- | --- |
| cranium |  |  |  |
| **PC1** | **1,75** | **39.8** | **< 0.001** |
| **PC2** | **1,75** | **53.3** | **< 0.001** |
| PC3 | 1,75 | 0.032 | 0.858 |
| PC4 | 1,75 | 0.011 | 0.918 |
| PC5 | 1,75 | 0.707 | 0.403 |
| mandible |  |  |  |
| **PC1** | **1,65** | **30.2** | **< 0.001** |
| **PC2** | **1,65** | **62.03** | **< 0.001** |
| PC3 | 1,65 | 2.22 | 0.141 |

References

Adams D, Collyer M, Kaliontzopoulou A, and Sherratt E. 2017. Geomorph: Software for geometric morphometric analyses. *R package version 3.0.5*.
